# Endoscopic Endonasal Resection of Symptomatic Rathke Cleft Cysts: Total Resection or Partial Resection

**DOI:** 10.3389/fneur.2021.701177

**Published:** 2021-09-24

**Authors:** Xiejun Zhang, Jihu Yang, Yan Huang, Yufei Liu, Lei Chen, Fanfan Chen, Guodong Huang

**Affiliations:** ^1^Department of Neurosurgery, Health Science Center, Shenzhen Second People's Hospital, Shenzhen University First Affiliated Hospital, Shenzhen, China; ^2^Department of Endocrinology, Health Science Center, Shenzhen Second People's Hospital, Shenzhen University First Affiliated Hospital, Shenzhen, China

**Keywords:** Rathke cleft cyst, endoscopy endonasal surgery, total resection, partial resection, cyst wall

## Abstract

**Objective:** Rathke cleft cysts (RCC) are benign sellar lesions, and endoscopic endonasal surgery (EES) for symptomatic RCC is becoming increasingly popular, but total resection or partial resection (TR or PR) of the cyst wall is still inconclusive. The aim of this study was to review the complications and clinical prognoses associated with total and partial resection of the cyst wall by EES.

**Methods:** We retrospectively analyzed a series of 72 patients with symptomatic RCC treated by EES from -January 2011 to June 2019 at Shenzhen University First Affiliated Hospital. For these 72 cases, 30 were treated with TR and 42 were treated with PR. Intra- and post-operative complications and clinical prognosis were investigated.

**Results:** All 72 patients underwent a pure EES. In the TR group, 10 patients (33.3%) had intraoperative cerebrospinal fluid leakage (CSF leak), three patients (10%) had postoperative CSF leak, eight patients (26.7%) had postoperative diabetes insipidus (DI), eight patients (26.7%) had postoperative electrolyte disturbance, and 12 patients (40%) had temporary hypopituitarism postoperatively. While in the PR group, three patients (7.1%) had intraoperative CSF leak, two patients (4.8%) had postoperative DI, three patients (7.1%) had postoperative electrolyte disturbance, four patients (9.5%) had temporary hypopituitarism postoperatively, and no cases experienced postoperative CSF leak. The intra- and post-operative complications were significantly higher in TR group then PR group (P _IntraoperativeCSFleak_ = 0.004, P _Post−operativeCSFleak_ =0.036, P _TransientDI_ = 0.008, P _Temporaryhypopituitarism_ = 0.002, P _Permanenthypopituitarism_ = 0.036, P _Electrolytedisturbance_ = 0.023). No significant differences in post-operative improvement and recurrence.

**Conclusions:** EES is a safe and effective approach for the treatment of symptomatic RCC. Complete sucking out the cyst contents and partial resection of the cyst wall may be sufficient for treatment, and total resection of the cyst wall is associated with a higher incidence of complications.

## Introduction

Rathke cleft cysts (RCC) are benign cystic lesions of the sellar and suprasellar region, which is believed to arise from the remnants of the Rathke's pouch (1). RCC are frequently asymptomatic. However, when the RCC grows enough to cause compression of the surrounding structures, the patient can experience headaches, pituitary dysfunction, and/or visual disturbances (2).

Surgery for symptomatic RCC is necessary, and transnasal sphenoidal surgery (TSS) is recommended as the first line therapy for symptomatic RCC (3). With the development of endoscopic endonasal surgery (EES), EES has become the main surgical method for RCC (4, 5). Although EES is a safe and effective approach for the treatment of symptomatic RCC, it remains controversial whether the cyst wall should be undergo radical interoperative resection. In this study, we retrospectively analyzed the clinical complications and clinical prognosis of different degrees of cyst wall resection.

## Materials and Methods

### Patients

This study was approved by the ethics committee of the Shenzhen Second People's Hospital (also named as Shenzhen University First Affiliated Hospital). In this retrospective clinical study, a series of 72 patients with symptomatic RCC treated by EES were enrolled from January 2011 to June 2019, and these patients were separated into two treatment groups according to the surgical record, the total resection (TR) group (30 cases) who were subjected to full resection of the cyst wall and complete removal of the cyst contents and the partial resection (PR) group (42 cases) who were subjected to PR of the cyst wall (without forcibly resecting the cyst wall adhering to the pituitary gland and pituitary stalk) and full removal of the cyst contents. All RCC cases were confirmed by pathological examination. For these 72 cases, we obtained the following information from the inpatient medical record: sex, age, symptoms, magnetic resonance imaging (MRI) data, pituitary function, intra- and post-operative complications, and clinical follow-up results.

### MRI Examination

All patients underwent dynamic contrast-enhancement MRI examination of the pituitary region and measurement of the maximum diameter of the cyst.

### Evaluation of Pituitary Function

The preoperative assessment of pituitary function was performed in each patient at admission, and postoperative evaluation of pituitary function was evaluated in each patient within 1 week after surgery. The pituitary function is assessed by measuring blood levels of hormones secreted by the pituitary gland and its target organs, including thyroid function, reproductive hormone, cortisol, adrenocorticotropic hormone, and growth hormone. Postoperative pituitary function can be divided into three categories: normal, temporary hypopituitarism, and permanent hypopituitarism. Temporary hypopituitarism was diagnosed according to temporary low blood levels of hormones secreted by pituitary that requires hormone replacement to maintain normal function but can be recovered within 6 months. Permanent hypopituitarism was defined when hormone replacement is required for more than 6 months.

### Surgical Procedures

**Figure 1:**
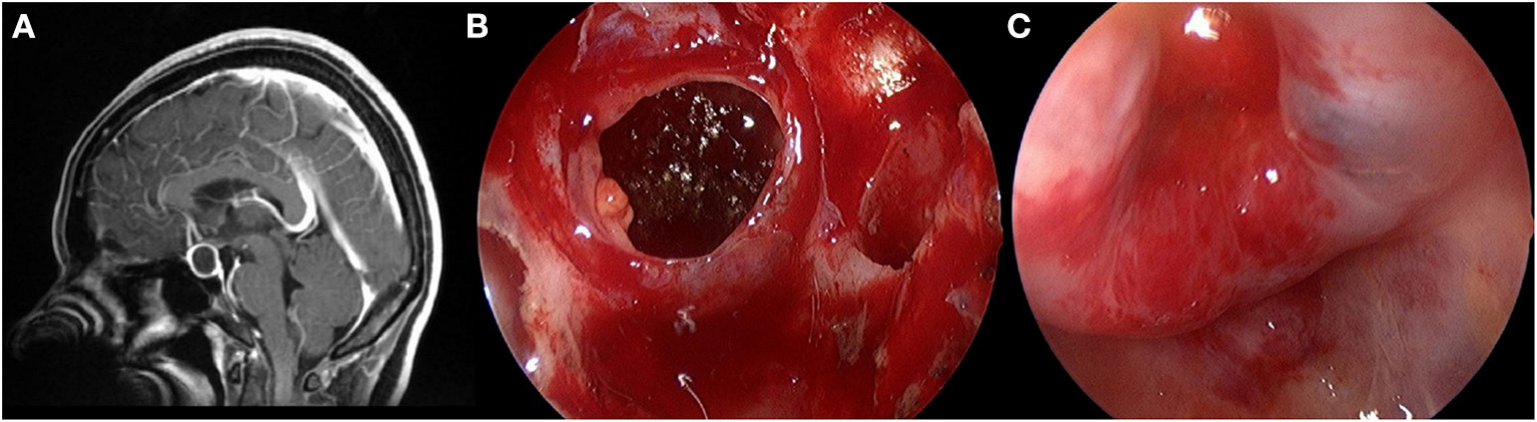
Total resection of the cyst wall structure. **(A)** Preoperative contrast-enhanced MRI showed a cystic mass within the sella. **(B)** The cyst wall is shown after the release of the cyst fluid. **(C)** Diaphragma sellae depression after radically resected the cyst wall.

**Figure 2:**
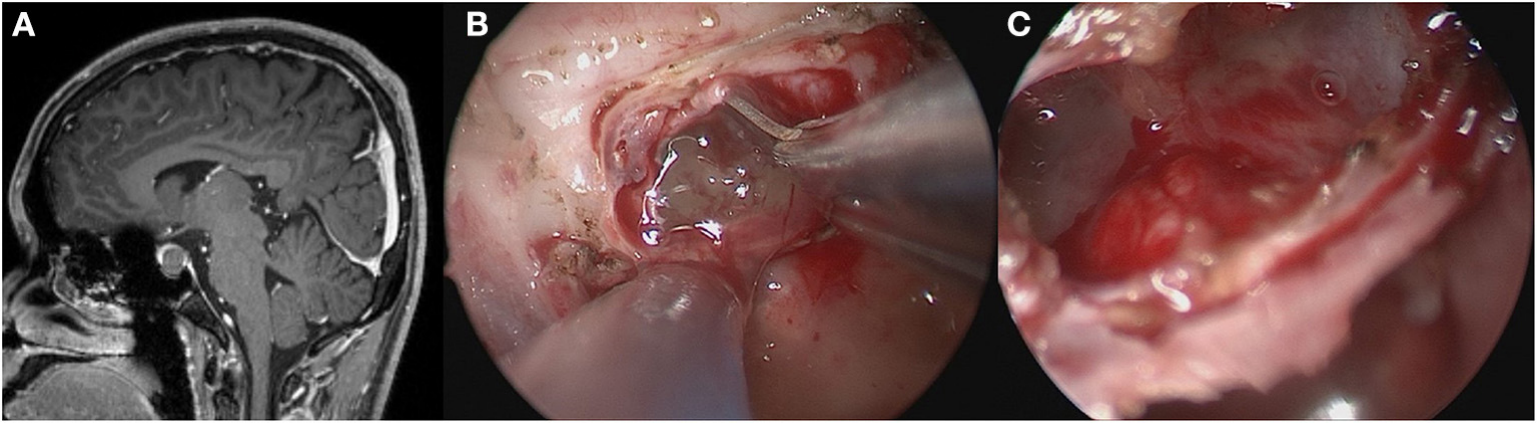
Partially resection of the cyst wall. **(A)** Preoperative contrast-enhanced MRI showed a cystic mass within the sella. **(B)** Exposed the gelatinous contents after opening the bottom of the saddle. **(C)** No further cleaning was done after the gelatinous contents were sucked out.

A standard EES was used to access the sphenoid sinus. Bilateral nostril manipulation was selected in most cases, while some cases were subjected to right nostril operation. The anterior wall and mucosa of the sphenoid sinus were removed. The bone structure in the sellar floor was identified, including bilateral carotid prominence, optic-carotid recess and clivus recess. The bone of sellar floor was removed with a rongeur and power drill. The dura mater was opened at the location close to the cyst and pituitary. The contents of the cyst flowed out naturally or were aspirated with an aspirator. In the TR group, the cyst wall structure was completely resected (Figure 1). In the PR group, only part of the cyst wall was removed for pathological examination, but did not forcibly resect the cyst wall adhering to the pituitary gland and pituitary stalk (Figure 2). If cerebrospinal fluid (CSF) leakage occurred intraoperatively, sellar reconstruction was performed with abdominal fat or quadriceps fascia, and then a small piece of artificial dura mater and external freeze-dried human fibrin adhesive was used to support the sellar floor.

### Pathological Examination

The cyst walls or cyst contents were fixed in 10% formalin and subjected to pathological examination. The obtained pathological specimens were embedded in paraffin, cut into sections, and stained with hematoxylin and eosin before microscopic examination by experienced pathologists.

### Postoperative Follow-Up

Clinical follow-up evaluations were first performed by a neurosurgeon to assess the resolution of clinical symptoms and pituitary function. The postoperative assessment of pituitary function was completed within 1 week after surgery and was repeated 4–8 weeks after surgery. Dynamic contrast-enhancement MRI examination of the pituitary region was reexamined 3–6 months postoperatively, and then once a year. When the patient's pituitary function was normal, follow-up with a neurosurgeon was continued. If hypopituitarism still existed, the patient was referred to an endocrinologist. If the postoperative symptoms recurred and follow-up MRI showed cyst recurrence, the patient was treated again.

### Statistical Analysis

Categorical variables were expressed as numbers (*n*) and percentages, and quantitative variables were expressed as means (average) ± standard deviations. Statistical analysis was conducted using Prism GraphPad (Version 5.0, La Jolla, California, USA) software. A student *t*-test was used to compare quantitative variables (age, cyst diameter, and follow-up period). Percentages of patients in the two groups were compared with the chi-square test. If the probability value was < 0.05, the difference was considered to be statistically significant.

## Results

### Clinical Features

A total of 72 patients with symptomatic RCC treated by EES from January 2011 to June 2019 at Shenzhen University First Affiliated Hospital were enrolled in this study, and all cases were confirmed by pathological examination. Among them, there were 30 males and 42 females, with an average age of 42 ± 12 years (range 18–74 years). There were 50 patients with headache, 16 patients with visual impairment, and six patients with pituitary dysfunction. Thirty patients were treated with TR, and 42 patients were treated with PR. The average age of the TR group was 42 ± 13 years, the average age of the PR group was 43 ± 12 years, and both groups had comparable manifestations (Table 1).

**Table 1:** Clinical presentations of 72 Patients with RCC.

	**TR group (30)**	**PR group (42)**	***P***
**General information**			0.808
Male	12	18	
Female	18	24	
**Mean age**	42 ± 13	43 ± 12	0.737
**Manifestations**			1.000
Headache	21	29	
Visual impairment	7	9	
Pituitary dysfunction	2	4	

### MRI Features of RCC

The signal intensity of the cysts were highly variable. Most of the cysts were on hyperintensity in T2-weighted image (83.3%), while about half of them were hypointensity on T1-weighted image (54.2%). Cyst enhancement was seen in 52.8% patients. The majority of the cysts (66.7%) were located in the sellar with suprasellar extension, with 29.2% cysts were entirely in the sellar region. Only three cysts (4.2%) were located entirely in the suprasellar region. The cyst diameters were measured preoperatively, ranging from 11 to 34 mm (average 17 mm) (Table 2).

**Table 2:** MRI features of 72 Patients with RCC.

	**TR group (30)**	**PR group (42)**	***P***
**MRI**			
T1-wighted image			0.643
Hypointensity	13	19	
Hyperintensity	14	16	
Isointensity	3	7	
T2-wighted image			0.538
Hypointensity	1	3	
Hyperintensity	26	34	
Isointensity	3	5	
Cyst enhancement	16	22	
**Location of cyst**			1.000
Sellar	8	13	
Sellar and suprasellar	21	27	
Suprasellar	1	2	
**Mean lesion size, mm**	17 ± 5	17 ± 6	1.000

### Intra- and Post-Operative Outcomes

In the TR group, 10 patients (33.3%) had intraoperative CSF leakage, three patients (10%) had postoperative CSF leakage, eight patients (26.7%) had postoperative diabetes insipidus (DI), eight patients (26.7%) had postoperative electrolyte disturbance, and 12 patients (40%) had temporary hypopituitarism postoperatively. In the PR group, three patients (7.1%) had intraoperative CSF leakage, two patients (4.8%) had postoperative DI, three patients (7.1%) had postoperative electrolyte disturbance, and four patients (9.5%) had temporary hypopituitarism postoperatively, while no cases experienced postoperative CSF leakage. The intra- and post-operative complications were significantly higher in TR group then PR group (P _IntraoperativeCSFleak_ = 0.004, P _Post−operativeCSFleak_ =0.036, P _TransientDI_ = 0.008, P _Temporaryhypopituitarism_ = 0.002, P _Permanenthypopituitarism_ = 0.036, P _Electrolytedisturbance_ = 0.023). The average follow-up was 56 ± 24 months for the TR group and 64 ± 22 months for the PR group, and there were no recurrence cases in both groups. No significant differences in post-operative improvement and recurrence (Table 3).

**Table 3:** Intra- and post-operative complications and follow-up outcomes.

	**TR group (30)**	**PR group(42)**	***P***
**Intra-operative CSF leak**	10	3	0.004
**Post-operative complications**			
CSF leak	3	0	0.036
Transient DI	8	2	0.008
Temporary hypopituitarism	12	4	0.002
Permanent hypopituitarism	3	0	0.036
Electrolyte disturbance	8	3	0.023
**Post-operative improvement**			1.000
Headache	16	23	0.905
Visual impairment	6	8	0.920
Pituitary dysfunction	1	4	1.000
**Recurrence**	0	0	1.000
**Mean follow-up, months**	56 ± 24	64 ± 22	

### Pathological Features

The cyst contents were yellow in 25 patients (34.7%) and white in 28 patients (38.9%). The morphology of the epithelium in most patients was simple cuboidal and simple columnar (84.7%) and there were nine patients (12.5%) with squamous metaplasia. Chronic inflammation were seen in 25% of the patients and cholesterol crystal were present in 29.2% of the specimens (Table 4).

**Table 4:** Cyst content and histopathological features of 72 Patients with RCC.

	**TR group (30)**	**PR group (42)**	***P***
**Color of content**			0.800
Yellow	10	15	
White	12	16	
CSF-like	5	4	
Brown	3	5	
Hemorrhagic	0	2	
**Histopathological**			
Epithelial lining			0.472
Simple	25	36	
Pseudostratified	1	1	
Squamous	4	5	
Chronic inflammation	7	11	0.783
Cholesterol crystal	8	13	0.693

### Follow-Up Outcomes

The overall follow-up time was 56 ± 24 months (ranging 23–96 months) for the TR group and 64 ± 22 months (ranging 23–98 months) for the PR group, and there was no recurrence in both groups (Table 4). The pituitary function of 16 patients with postoperative hypopituitarism were recovered during the follow-up period, and five patients with preoperative hypopituitarism was recovered back to normal pituitary function after surgery. Two patients in TR group experienced permanent hypopituitarism and thus oral hormone replacement therapy could not be discontinued.

## Discussion

RCC originates from the remnants of embryonic Rathke's pouch and is usually located between the neurohypophysis and the adenohypophysis (1, 2). RCCs are often started asymptomatic, and with the growth of the lesions, they can oppress the surrounding structures, including the diaphragma sellae, optic chiasm and pituitary gland, thus leading to headaches, visual impairment and pituitary dysfunction (2).

Typical symptomatic RCCs appear between the ages of 40 and 50, with a slightly preponderance in females (3, 6, 7). Headache, visual impairment and pituitary dysfunction were the three major manifestations of symptomatic RCC, with the incidence of headache ranging from 33 to 100% (8, 9), visual impairment ranging from 16 to 47%, and pituitary dysfunction ranging from 12 to 58.3% (8–10). In this study, headache was the most common clinical symptom (69.4%), followed by visual impairment (22.2%) and pituitary dysfunction (8.3%).

Surgery is an effective method to alleviate the mass effect of RCC, and TSS is recommended as the first line therapy for symptomatic RCC (3). With the development of neuroendoscopic technology, the endoscopic treatment of RCC has been recognized by more and more neurosurgeons, and EES has become the preferred surgical approach for RCC (4, 5). According to earlier literature, headache and visual impairment are usually significantly improved postoperatively (11–13). Our results showed that preoperative headache and visual impairment were resolved in 78.0 and 87.5% of patients, respectively.

EES is a safe and effective approach for the treatment of symptomatic RCC, but it is still controversial whether the cyst wall should undergo radical intraoperative resection or not. RCC recurrence has been an important challenge for EES approach, with the rates ranging from 3 to 33% (3). Several previous studies have shown that the recurrence of RCC is related to the degree of cyst wall resection (14, 15). However, increasing evidence has demonstrated that radical resection of the cyst wall is associated with a higher incidence of complications, and attempted radical resection does not appear to reduce the overall rate of recurrence (6, 9, 11, 16). Consistent with previous studies, our data here show that radical resection is associated with increased intra- and post-operative complications, including CSF leakage, postoperative DI, temporary hypopituitarism and electrolyte disturbance. Of note, in this study, no recurrence has been observed within an average follow-up period of 61 months for both radical resection and partial resection. Previous retrospective studies have suggested that the recurrence of RCC correlates with several factors, including the pattern of cyst wall enhancement, the residual cyst on MRI postoperative, the pathological features, and fat graft insertion, but not with aggressive excisions of the cyst wall (3, 6, 17, 18). It is worth pointing out that no recurrence in our study may be due to a relatively short follow-up period.

## Conclusion

Taken together, our data support that the strategy of complete aspiration of the cyst contents and partial resection of the cyst wall is effective in treating symptomatic RCC. Aspiration of the cyst contents can achieve adequate decompression, while partial resection of the cyst wall can minimize the incidence of intra- and post-operative complications, including CSF leakage, pituitary dysfunction, electrolyte disturbance and DI.

## Data Availability Statement

The original contributions presented in the study are included in the article/supplementary material, further inquiries can be directed to the corresponding authors.

## Ethics Statement

The studies involving human participants were reviewed and approved by Research Ethics Committee of the First Affiliated Hospital of Shenzhen University. The patients/participants provided their written informed consent to participate in this study.

## Author Contributions

XJZ and GDH conceived and designed the experiments. XJZ drafted the manuscript. JHY, YH and YFL analyzed the data. LC and FFC helped to collect clinical data. All authors read and approved the final manuscript.

## Conflict of Interest

The authors declare that the research was conducted in the absence of any commercial or financial relationships that could be construed as a potential conflict of interest.

## Publisher's Note

All claims expressed in this article are solely those of the authors and do not necessarily represent those of their affiliated organizations, or those of the publisher, the editors and the reviewers. Any product that may be evaluated in this article, or claim that may be made by its manufacturer, is not guaranteed or endorsed by the publisher.

## Abbreviations

RCC: Rathke cleft cysts
EES: Endoscopic endonasal surgery
TR: Total resection
PR: Partial resection
CSF leak: Cerebrospinal fluid leakage
DI: Diabetes insipidus
MRI: Magnetic resonance imaging.
